# Comparative Analyses of Unsupervised PCA K-Means Change Detection Algorithm from the Viewpoint of Follow-Up Plan

**DOI:** 10.3390/s22239172

**Published:** 2022-11-25

**Authors:** Deniz Kenan Kılıç, Peter Nielsen

**Affiliations:** Department of Materials and Production, Aalborg University, 9220 Aalborg, Denmark

**Keywords:** change detection, unsupervised learning, remote sensing, synthetic aperture radar, SAR image change detection, SAR images, follow-up plan, principal component analysis, k-means clustering

## Abstract

In this study, principal component analysis and k-means clustering (PCAKM) methods for synthetic aperture radar (SAR) data are analyzed to reduce the sensitivity caused by changes in the parameters and input images of the algorithm, increase the accuracy, and make an improvement in the computation time, which are advantageous for scoring in the follow-up plan. Although there are many supervised methods described in the literature, unsupervised methods may be more appropriate in terms of computing time, data scarcity, and explainability in order to supply a trustworthy system. We consider the PCAKM algorithm, which is used as a benchmark method in many studies when making comparisons. Error metrics, computing times, and utility functions are calculated for 22 modified PCAKM regarding difference images and filtering methods. Various images with different characteristics affect the results of the configurations. However, it is evident that the PCAKM becomes less sensitive and more accurate for both the overall results and image results. Scoring by utilizing these results and other map information is a gap and innovation. Obtaining a change map in a fast, explainable, more robust and less sensitive way is one of the aims of our studies on scoring points in the follow-up plan.

## 1. Introduction

Change detection for temporal differential images is the implementation of an the algorithm/method to detect the changes that have occurred between two images obtained at different times from the same sensor, platform, and location. In other words, it is a process to divide the map into regions that are changed and unchanged.

Change detection algorithms are used in several areas such as video surveillance, remote sensing, medical diagnosis and treatment, civil infrastructure, underwater sensing, and driver assistance systems [1]. Different types of systems in remote sensing and aerial photography are used to detect changes between the scenes of the same location acquired at different times, which is also called remote sensing change detection [2]. Depending on the sensors, systems used, and the time–frequency of the images obtained, different tasks are assigned and executed. Such changes can trigger follow-up activities to determine the cause or type of change, such as triggering additional image requests [3,4], direct actions such as search and rescue missions [5], or influencing decisions made in the area, e.g., threat avoidance [6]. In all of these cases, quick, precise, and interpretable change detection is critical to deriving timely information and properly reacting in the subsequent follow-up. A synthetic aperture radar (SAR) sensor is extensively used in numerous areas [7] to obtain change maps since it is not affected by the weather, light, or flight altitude [8,9]. These images typically form the function for actions such as those listed above. Speckle noise [10,11,12], fuzzy edge of changed regions [12], and limited datasets [12] are the main challenges for the change detection of SAR images.

Future changes that are likely to occur can be predicted using spatial and temporal dynamics. Therefore, the follow-up information acquisition can be streamlined, by creating the foundation for the follow-up plan by scoring the predicted change detection map [13]. Follow-up activities require the consideration of the estimated change map’s accuracy and computation time. Most follow-up planning may not allow for the large amounts of data that are necessary for supervised learning. Due to this, the follow-up planning benefits the adoption of unsupervised techniques that do not need training data and have quick computation times. In addition, classical methods generally provide more transparency, explainability, and interpretability than more complex ones do [14]. These properties support the system’s trustworthiness [15,16,17], which is significant for any response to the detected change.

In this study, the change maps of different satellite images are calculated using the unsupervised change detection algorithm proposed by Celik [18] and called principal component analysis and k-means clustering (PCAKM). In situations where the follow-up plan needs to be made in short periods (such as disaster response, etc.) and training data are lacking, it is more appropriate to use unsupervised methods instead of supervised methods. In addition, it is not necessary for unsupervised change detection methods to specifically identify the kinds of changes in land use or cover that have occurred in the area of interest [19]. Depending on the change in the parameters used in PCAKM, the calculation times and performance results of the obtained change detection alter. Moreover, altering the inputs for Celik’s algorithm affects results notably.

We produce several configurations as modified PCAKMs using different filters and DIs. The performance results obtained based on modified versions of Celik’s algorithm are compared and examined to understand whether they are suitable for generating scores to form the foundation for planning follow-up detailed investigations or responses. As a result of these investigations, we seek to answer the following questions:

- Is it possible to decrease sensitivity or increase consistency?
- Is it possible to decrease computing time without decreasing accuracy?

These questions are critical to obtaining a modified method that is less affected by different PCAKM algorithm parameters and input image characteristics. The way to achieve this is to increase the average performance and reduce the variance of the results obtained. On the other hand, providing a decrease in computing time is important in real-time tasks. Change maps with a less sensitive method will then be input into the scoring stage for a follow-up plan. Change maps with lower error rates and variance will help the selection of the areas of interest (AOIs) generating the points of these AOIs and scoring. A gap and innovation in the follow-up plan is scoring using change map results and other map information. We aim to contribute to scoring points in the follow-up plan by focusing on obtaining the change map in a quick, explainable, more accurate, and less sensitive manner.

The paper is organized as follows. Section 2 provides a related literature review and the methods used in this paper. Section 3 explains the data, configurations, and performance metrics used in experiments, and shows the results. Comments and discussions on the results are included in Section 4. Finally, the paper is concluded in Section 4.2.

## 2. Methods

To find answers to the questions in Section 1, we make comparative analyses by performing unsupervised change detection and performance evaluation. Performance measurements were carried out using the unsupervised learning method for the change detection part. The method includes principal component analysis (PCA) and k-means clustering methods as described in [18].

The PCAKM method proposed by Celik [18] has been used as a benchmark comparison in many unsupervised and supervised SAR image change detection studies and continues to be used in the state-of-the-art research. Li et al. [20] compared PCAKM, Markov random field fuzzy c-means (MRFFCM), Gabor fuzzy c-means (GaborFCM), and Gabor two-layer classifier (GaborTLC). They determined that the Kappa coefficient (KC) difference between PCAKM and other methods for the benchmark data is at most 2.67%. Gao et al. [13] proposed deep semi-non-negative matrix factorization (NMF) and a singular value decomposition network to compare with PCAKM, MRFFCM, GaborTLC, and deep neural networks with MRFFCM (D_MRFFCM). KC differences between PCAKM and methods that give better results for the three benchmark data are less than or equal to 6.77%, 7.57%, and 3.3%, respectively. In addition, PCAKM has a higher KC than MRFFCM and GaborTLC for two out of three data. Gamma deep belief network (gΓ-DBN) is proposed by Jia and Zhao [12] for comparison with PCAKM, convolutional-wavelet neural network (CWNN), deep belief network (DBN), and joint deep belief network (JDBN). According to their experimental results, PCAKM shows better performance than CWNN and DBN for one of the benchmark datasets in terms of KC. When all images are examined, improvements in KC for PCAKM are less than or equal to 7.98%. Wang et al. [21] presented a graph-based knowledge supplement network (GKSNet) to match against PCAKM, a neighborhood-based ratio and extreme learning machine (NR-ELM), Gabor PCA network (GaborPCANet), local restricted convolutional neural network (LR-CNN), transferred multilevel fusion network (MLFN), DBN, and deep cascade network (DCNet). In their study, the KC/F1-measure enhancements for PCAKM are less than or equal to 10.71%/9.42%, 2.75%/2.2%, 19.21%/22.57%, and 11.43%/9.75% for four different benchmark data, respectively. Even though the supervised methods provide these improvements in terms of accuracy, their run-time results show that there are reasonably high differences between PCAKM and other methods. The average run-times in seconds for PCAKM, NR-ELM, GaborPCANet, LR-CNN, MLFN, DBN, DCNet, and GKSNet are 2.3, 22.5, 442.8, 282.6, 187.6, 474.1, 509.6, and 144.92, respectively [21].

Considering its features such as being fast, not requiring learning data, and having a simple algorithm, we selected PCAKM as a benchmark method. It shows promising results for both unsupervised [13,18,20] and supervised methods [12,21].

Speckle is a type of grainy noise that occurs naturally in active radar, SAR, medical ultrasound, and optical coherence tomography images, and decreases their quality. Images of the same region taken at different times have different levels of speckle. Speckling creates difficulty in distinguishing opposite classes [22] since it increases the overlap of opposite-class pixels in the histogram of difference images. On the other hand, there is competitive interaction between altered regions and background regions due to a lack of past information, resulting in a fuzzy edge in the changed region that is difficult to discern. Another challenge is the lack of data, which is an issue for supervised learning.

Noise may develop as a result of the system’s construction, illumination conditions, and image acquisition process. Numerous methods were proposed for speckle reduction or despeckling. Speckle reduction filters are classified as non-adaptive and adaptive filters [23]. Mean and median filtering methods are examples of non-adaptive techniques. On the other hand, Lee, Frost, Kuan, and G-MAP are adaptive filter examples. Qiu et al. [24] claimed that none of these filters consistently outperform others, in principle. Each filter has particular advantages and disadvantages according to the data. For this reason, choosing a more stable and consistent filter is important.

Moreover, speckling reduction techniques are categorized as the spatial domain, transform domain (or wavelet domain), non-local filtering, and total variational [23]. Specifically, anisotropic diffusion, bilateral filter (BF), fast non-local means filter (FNLMF), and guided filter (GF) are some other filters to reduce speckle noise [25]. Choi and Jeong [25] state that BF and the non-local mean filter (NLMF) have a low speckle noise reduction performance. In addition, non-linear methods such as BF and NLMF have poor computational time performance [25]. Partial differential Equation (PDE)-based algorithms including AD and adaptive window anisotropic diffusion also have a weak performance on speckle noise removal [25]. Some other conventional filtering methods such as discrete wavelet transform (DWT), Bayesian multiscale method in a non-homomorphic framework, and expectation maximization DWT perform poorly in terms of speckle noise removal, edge information preservation, and computing complexity [25]. We test the edge-protecting GF method, which has low computational complexity among the speckle noise elimination techniques considering the performance results for the SAR images in [25]. We also used the NLMF and BF methods to acquire the performance characteristics mentioned above.

In this study, we compared the PCAKM with its modified versions (different combinations of difference images and filters) in terms of accuracy and time performance. We consider whether there is a modified method with less sensitivity and higher accuracy for the change map to be used in any follow-up plan.

### 2.1. Original PCA K-Means Algorithm

**Figure 1:**
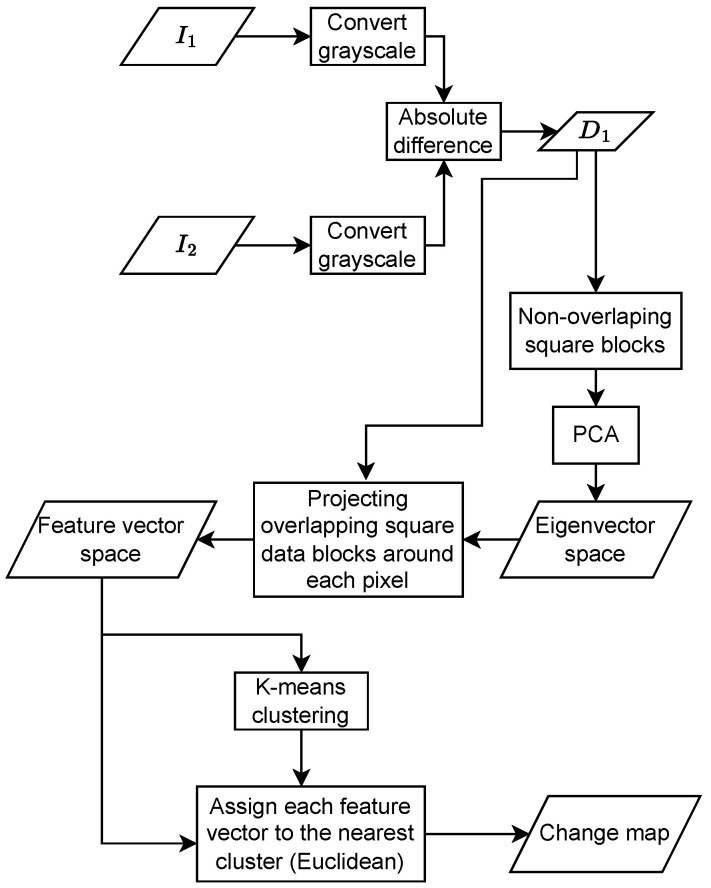
Unsupervised Change Detection Algorithm Proposed by Celik [18].

The flow of the proposed original method is given in Figure 1 [18]. PCA and k-means methods [18] are utilized for the change detection part.

Firstly, the input image pairs $I_{1}$ and $I_{2}$ are converted into grayscale images. Then, the absolute difference image for the given image pair is calculated as

(1) $$D_{1}=\left|I_{1}-I_{2}\right|.$$

Afterward, $D_{1}$ is divided into bs×bs non-overlapping blocks where bs is the length of one side of square blocks. After converting these blocks into row vectors, PCA is applied to these vector sets to obtain the orthonormal eigenvectors. In the next step, the feature vector space is created by projecting bs×bs overlapping blocks around each pixel onto the eigenvector space. Feature vector space is input for the k-means algorithm to get the change map. Using the k-means algorithm, the feature vector space is grouped into clusters. Then, each pixel is assigned to a cluster in a way that minimizes the distance between its feature vector and the cluster’s mean vector. Briefly, we used two parameters bs and *k* as block width and cluster number, respectively. In Section 3, bs is between 2 and 8, whereas *k* is 2 and 3 for each image pair.

### 2.2. Other Difference Image Methods

The log-ratio difference image method, which is given in Equation (2), is utilized in many studies to reduce the multiplicative distortion effects of noise caused by speckle [10]. Moreover, Zhao et al. [26] produced the difference image via image regression as given in Equation (3) to avoid problems such as atmospheric condition changes, illumination variations, and sensor calibration [27]. The image regression method enhances the performance of the difference image, which is observed from direct subtracting. However, both the log-ratio and absolute log-ratio methods still do not perform well enough to eliminate speckle noise if the input becomes low-quality [10,27].

**Definition** **1**  (Log-ratio difference image). *Log-ratio image is the logarithmic transform of the image pair’s division as*

(2) $$D_{2}=f\left(\frac{I_{1}}{I_{2}}\right),$$

*where f=c∗log((1+p),10), c≈105 for all pixels $p\in \frac{I_{1}}{I_{2}}$.*

**Definition** **2**  (Absolute log-ratio difference image). *It is the absolute value of the log-ratio calculation as*

(3) $$D_{3}=\left|f\left(\frac{I_{1}}{I_{2}}\right)\right|.$$

Zhang et al. [10] stated that the SAR images are contaminated by speckle noise, which has the multiplicative Goodman’s model. The Nakagami distribution in Equation (4) is then used to represent the independently and identically distributed pixel amplitudes. The Nakagami distribution is

(4) $$p\left(I_{s}|R_{s}\right)=\frac{2L^{L}}{\Gamma \left(L\right){\left(R_{s}\right)}^{L}}I_{s}^{2L-1}\exp\left(-\frac{LI_{s}^{2}}{R_{s}}\right),$$

where $R_{s}$ and $I_{s}$ are the reflectivity and pixel amplitudes in site *s*, respectively. Moreover, *L* is the equivalent number of looks, which is a parameter of multi-look SAR images, and represents the amount of averaging done to the SAR measurements both during the creation of the data and, on occasion, even after [28]. After several calculations to which Bayesian decision theory was applied, the difference image is given as in Equation (5), where it considers the knowledge that the speckles follow the Nakagami distributions.

**Definition** **3**  (Nakagami log-ratio (NLR) difference image). *It is a modified version of the log-ratio difference image given as*

(5) $$D_{4}=f\left(\frac{I_{1}}{I_{2}}+\frac{I_{2}}{I_{1}}\right).$$

*Its absolute version can be written as*

(6) $$D_{5}=\left|f\left(\frac{I_{1}}{I_{2}}+\frac{I_{2}}{I_{1}}\right)\right|.$$

**Definition** **4**  (Modified NLR difference image 1). *In this version of the NLR difference image, we use the squared values of each image given as*

(7) $$D_{6}=f\left(\frac{I_{1}^{2}}{I_{2}^{2}}+\frac{I_{2}^{2}}{I_{1}^{2}}\right).$$

*Its absolute value is*

(8) $$D_{7}=\left|f\left(\frac{I_{1}^{2}}{I_{2}^{2}}+\frac{I_{2}^{2}}{I_{1}^{2}}\right)\right|.$$

**Definition** **5**  (Modified NLR difference image 2). *For this modified version of the NLR difference image, squares of each division are added to the NLR difference image itself as*

(9) $$D_{8}=f\left(\frac{I_{1}}{I_{2}}+\frac{I_{2}}{I_{1}}+\frac{I_{1}^{2}}{I_{2}^{2}}+\frac{I_{2}^{2}}{I_{1}^{2}}\right).$$

*The absolute value of it is*

(10) $$D_{9}=\left|f\left(\frac{I_{1}}{I_{2}}+\frac{I_{2}}{I_{1}}+\frac{I_{1}^{2}}{I_{2}^{2}}+\frac{I_{2}^{2}}{I_{1}^{2}}\right)\right|.$$

**Definition** **6**  (Improved ratio and log improved ratio difference image [29,30]). *The improved ratio and its log transform version are given in Equations (11) and (12), respectively.*

(11) $$D_{10}=1-\frac{\min\{I_{1},I_{2}\}}{\max\{I_{1},I_{2}\}},$$

(12) $$D_{11}=f\left(1-\frac{\min\{I_{1},I_{2}\}}{\max\{I_{1},I_{2}\}}\right).$$

### 2.3. Non-Local Means Denoising

Basically, the color of a pixel is changed to an average of the colors of nearby pixels by non-local means denoising (NLMD) [31]. Since there is no justification for the closest pixels to a given pixel to be even close, it searches across a sizable chunk of the image for every pixel that resembles the pixel to be denoised. There are three parameters such as *h*, *templateWindowsSize (tws)*, and *searchWindowsSize (sws)*. The first one regulates the filter strength. If it is increased, then it removes the noise more precisely but removes the image details as well and vice versa. The *tws* parameter is the template patch’s size in pixels, which is utilized to calculate weights. Lastly, *sws* is the window’s size in pixels that is applied to estimate the weighted average for a specific pixel. We used OpenCV’s recommended values for the last two parameters as 7 and 21, respectively. On the other hand, for *h*, we used 20 since SAR images contain a high degree of noise.

### 2.4. Bilateral Filter

In addition to using a (multiplicative) Gaussian filter component that is based on pixel intensity differences, the bilateral filter (BF) also employs a Gaussian filter in the space domain. Only pixels that are “spatial neighbors” are taken into account for filtering, owing to the Gaussian function of space. On the other hand, the Gaussian component used in the intensity domain makes sure that only the pixels with intensities close to the core pixel are taken into account when computing the blurred intensity value. BF is a method that preserves the edge information. We used 10 for the parameter *denoisingWindowsize (dws)*, which is larger than the default value 3, which is similar to NLMD, and we consider that the SAR image has substantial noise.

### 2.5. Guided Filter

A guided filter is a smoothing light filter that preserves the edges. It filters out noise or texture while keeping sharp edges, just like a bilateral filter [32,33]. The GF is defined by the following Equations (13)–(15) as

(13) $$a_{k}=\frac{\frac{1}{\left|w\right|}\sum_{i\in w_{k}}I_{i}p_{i}-\mu_{k}{\bar{p}}_{k}}{\sigma_{k}^{2}+\epsilon },$$

(14) $$b_{k}={\bar{p}}_{k}-a_{k}\mu_{k},$$

(15) $$q_{i}={\bar{a}}_{i}I_{i}+{\bar{b}}_{i},$$

where $\left(a_{k},b_{k}\right)$ are linear coefficients for a linear transform of the guidance image *I* at a pixel *i* with the input image *p* and supposed to be constant in a window $w_{k}$ (square window of a radius *r*) centered at the pixel *k*. Furthermore, $\mu_{k}$ and $\sigma_{k}^{2}$ are the mean and variance of *I* in $w_{k}$, |w| is the numbers of pixels in $w_{k}$, ${\bar{p}}_{k}=\frac{1}{\left|w\right|}\sum_{i\in w_{k}}p_{i}$ is the mean of *p* in $w_{k}$, and ϵ is a regularization parameter penalizing large $a_{k}$. Moreover, ${\bar{a}}_{i}=\frac{1}{\left|w\right|}\sum_{k\in w_{i}}a_{k}$ and ${\bar{b}}_{i}=\frac{1}{\left|w\right|}\sum_{k\in w_{i}}b_{k}$ are the overall average coefficients for windows that overlap with *i* where $q_{i}$ is the filtering output at a pixel *i*.

### 2.6. Truncated Singular Value Decomposition

Truncated singular value decomposition (TSVD) is a reduced rank approximation to any matrix A by selecting the first major singular values. We determine the subset of full components via the percentage of the total variance. Therefore, we utilize the var parameter that is a threshold for total variance. The reason for applying this method is to assess whether we can reduce the time performance without much loss of accuracy.

## 3. Experiments

### 3.1. Data

Details of data used in experimental results are given in Table 1. For each image pair, there is a ground truth image for the change between the two images. The ground truth images are used to generate the confusion matrices and calculate the performance metrics mentioned above.

In Table 2, noise variance values based on the method in [34] are given.

**Figure 2:**
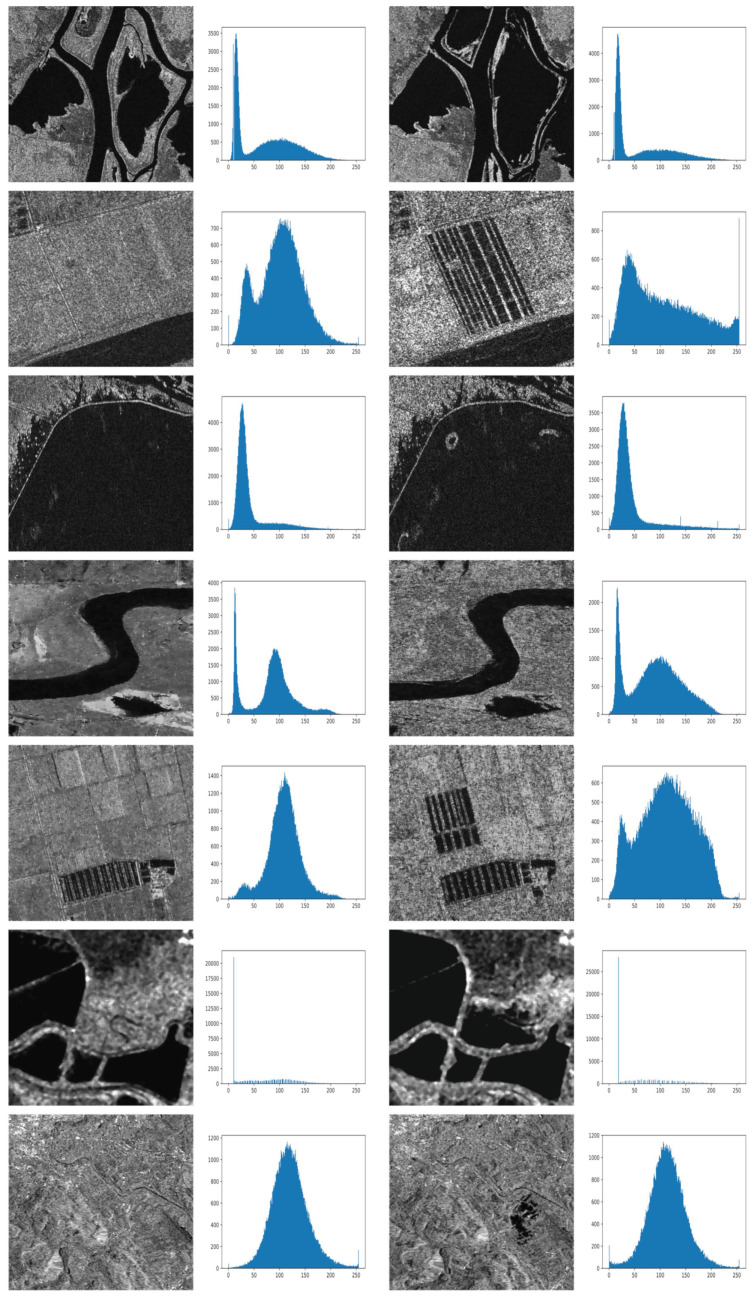
Image pairs and their histograms.

In Figure 2, all images with histograms are demonstrated.

**Figure 3:**
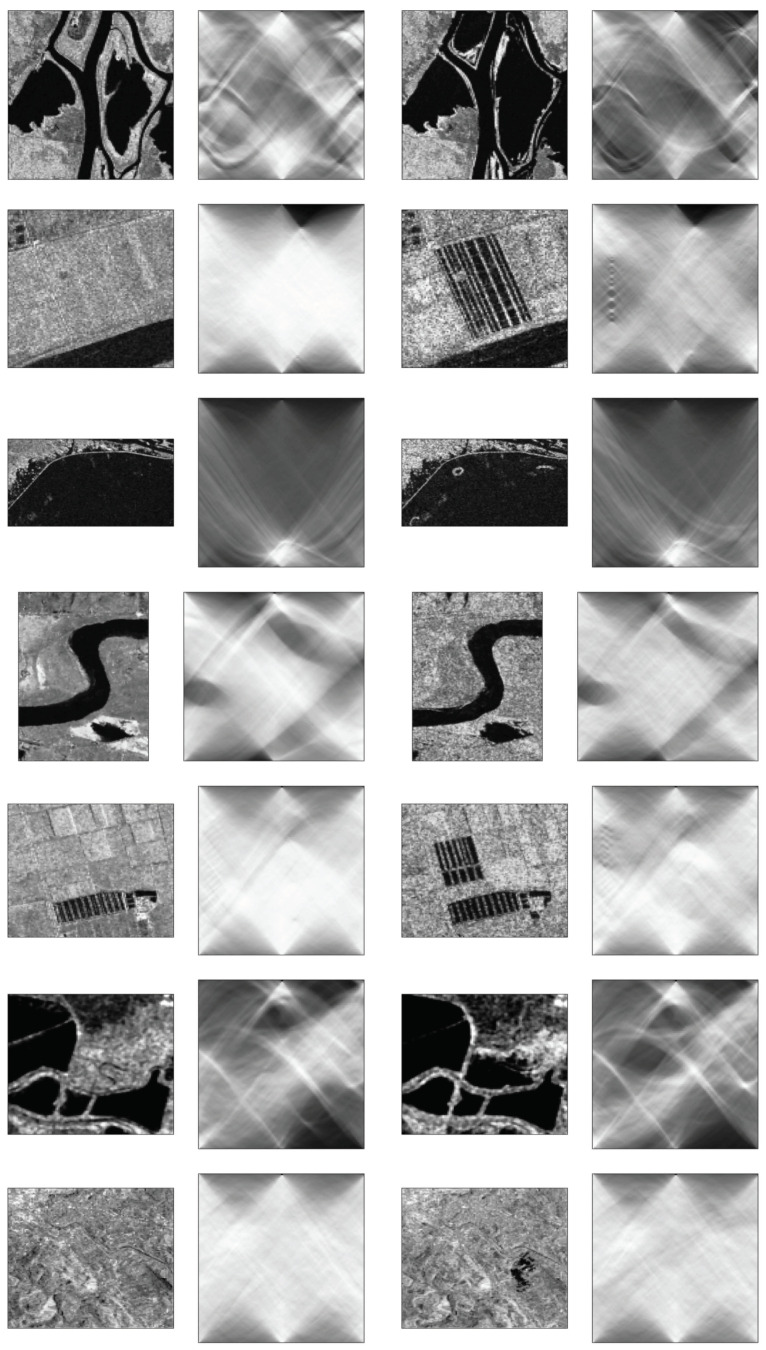
Image pairs and their Radon transforms.

In Figure 3, Radon transforms between 0 and 180 degrees are illustrated. Radon transforms, which are also called sinograms, calculate image matrix projections over predetermined directions where lighter tones are more intense.

It is apparent that there are different characteristics not just among each image pair but also between some image pairs across the set, as shown in Table 2, Figure 2 and Figure 3.

### 3.2. Configurations

There are seven SAR image pairs and seven ground truths for change maps as data. We utilized 22 different configurations among which one is the original paper [18] as a benchmark and the others are modified versions of the original method. For each configuration, there are 98 (7 × 14) change detection results for each performance metric since we have two main parameters *block size* and *number of clusters*, which take values in the ranges of 2–8 and 2–3, respectively. We calculate each change detection result 1000 times and then obtain the minimum, maximum, and average calculation times. The accuracy results do not change for these 1000 experiments since all 22 configurations have deterministic skeletons.

All configurations are given in Table 3 with configuration numbers. Configurations containing more than one method are written according to the order of their implementation. The PCAKM algorithm is used after applying the written methods for any configuration. We select the radius of the square window (*r*) for GF as the *block size* (bs) parameter of the PCAKM algorithm. Explanations for other parameters in Table 3 are given in Section 2.

### 3.3. Performance Metrics

After calculating the change maps, performance metrics are estimated using the confusion matrix that is given in Table 4.

Below are formulations for performance metrics using the true positive (*tp*), false positive (*fp*), false negative (*fn*), and true negative (*tn*) in the confusion matrix as

- Percentage correct classifications: pcc=(tp+tn)/n
- Kappa coefficient: kc=(pcc−p)/(1−p), where $p=\frac{\left(tp+fp)\times (tp+fn)+(fn+tn)\times (tn+fp\right)}{n^{2}}$
- Precision: prec=tp/(tp+fp)
- Recall: recall=tp/(tp+fn)
- F-measure: fmeas=2×prec×recall/(prec+recall)

where n=tp+tn+fp+fn.

We use *Kappa coefficient* and *f-measure* as accuracy calculations. The range of the former is [−1,1] and the latter has a range of [0,1]. For both metrics, a higher value means better accuracy.

On the other hand, we estimate the utility functions by employing *Kappa coefficient*, *f-measure*, and average computing times. For each image pair, we have two utility values as

(16) $$U1_{ij}=\mu_{ij1}+\mu_{ij2}-\sigma_{ij1}^{2}-\sigma_{ij2}^{2},$$

(17) $$U2_{ij}=\left(\mu_{ij1}+\mu_{ij2}-\sigma_{ij1}^{2}-\sigma_{ij2}^{2}\right)/{\bar{t}}_{ij},$$

where $\mu_{ij1}$ is the average of *Kappa coefficient* values, $\mu_{ij1}$ is the average of *f-measure* values, $\sigma_{ij1}^{2}$ is the variance of *Kappa coefficient* values, $\sigma_{ij2}^{2}$ is the variance of *f-measure* values, ${\bar{t}}_{ij}$ is the mean of average computing times, *i* is the image pair number, and *j* is the configuration number for i=1,…,7 and j=1,…,22. For each configuration, we have 14 results since we utilize the parameters *block size* and *number of clusters*, which take values in the ranges 2–8 and 2–3, respectively. Then, we use these 14 values for mean and variance calculations. On the other hand, we have 14 different average time calculations and each parameter pair result is calculated 1000 times. Then, we take the average of these 1000 calculation times and estimate the mean of 14 average calculation times.

In addition to the U1 and U2 utility values, we calculate the following utility values for overall images in a single configuration as

(18) $$U3_{k}=\mu_{k1}+\mu_{k2}-\sigma_{k1}^{2}-\sigma_{k2}^{2},$$

(19) $$U4_{k}=\left(\mu_{k1}+\mu_{k2}-\sigma_{k1}^{2}-\sigma_{k2}^{2}\right)/{\bar{t}}_{k},$$

where $\mu_{k1}$ and $\mu_{k2}$ are the average *Kappa coefficient* value and the average *f-measure* value of all 98 results (14 parameters combination for seven images), $\sigma_{k1}^{2}$ and $\sigma_{k2}^{2}$ are the average variances of seven different image variance results for each configuration, ${\bar{t}}_{k}$ is the mean of seven images’ average time computations (each image has 14 different averaged time results for 1000 experiments), and *k* is the configuration number for k=1,…,22. Since, as we mentioned in Section 3.1, each image pair has different characteristics according to noise variances, histograms, and Radon transforms, we take the average of seven different image variance results for each configuration.

### 3.4. Results

**Figure A1:**
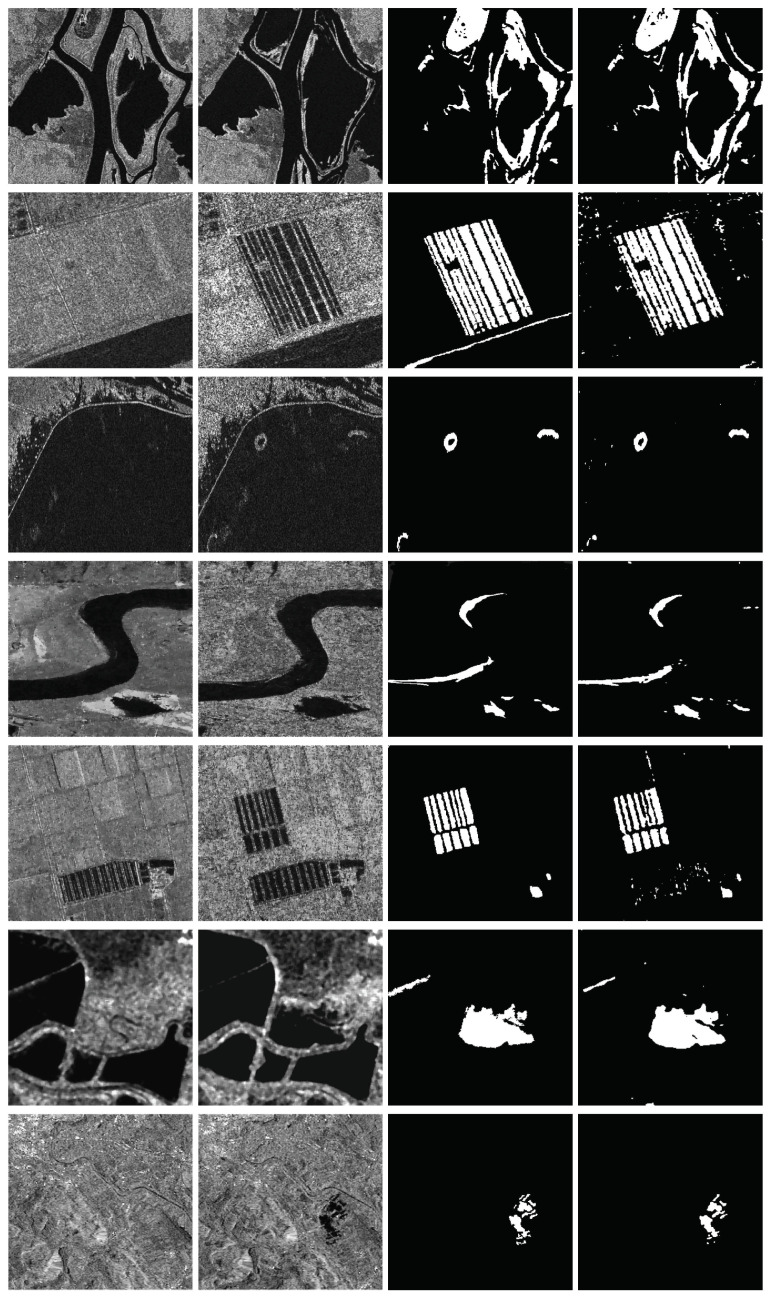
First two columns display SAR image pairs, third column shows ground truth change map, and fourth column illustrates predicted best change map results.

The best and the worst results and the mean and variance for error metrics (*kc* and *fmeas*) of 22 configurations, are given in Table A1, Table A2, Table A3, Table A4, Table A5, Table A6 and Table A7 for each image pair, respectively. The *bs* and *c* demonstrate the *block size* and the *number of clusters* parameters. Furthermore, the “*No*” columns in the tables state configuration numbers. We calculate the U1 and U2 utility values in these tables by utilizing each configuration’s average computing time. Image pairs, ground truth images, and the best change map result for all images are given in Figure A1 in Appendix B. The first two columns contain the image pairs. The third and fourth columns are the ground truths and best change map results, respectively.

Based on the image results given in Appendix A, Table 5 shows the order of configurations from largest to smallest utility values.

On the other hand, Table 6 presents the overall mean and variance of error metrics for each configuration with average computing times regarding the configuration numbers. We calculated U3 and U4, where the mean and variance values are the average values of seven different image results. Bold values show the highest mean and lowest variance estimations for error metrics.

Table 7 demonstrates the ranking of U3 and U4 values in terms of configuration numbers.

## 4. Discussion

### 4.1. Image-Based Results

D3 (config. 8), D5 (config. 14), D7 (config. 17), and D9 (config. 20) are the absolute values of D2 (config. 2), D4 (config. 9), D6 (config. 15), and D8 (config. 18), respectively. If we look at Table A1, Table A2, Table A3, Table A4, Table A5, Table A6 and Table A7, it is evident that the absolute versions of difference images do not increase the mean accuracy values for any configurations and images. There is no systematic decrease or increase for variance and average time performances as well.

We check the configuration pairs 2&3, 9&11, and 9&12 to observe the effects of TSVD. Applying TSVD decreases the average computing times for the Ottawa, Yellow River 2, Yellow River 4, San Francisco, and Bern datasets. It increases the mean values for Ottawa and the Yellow River 4, decreases the variance for Ottawa and Yellow River 2, and increases variance for Yellow River 3. Otherwise, increases and decreases show variability.

Furthermore, if we consider configuration pairs 2&4, 9&10, 15&16, and 18&19, utilizing GF increases the average computing times for all images. However, there is no regular increase and decrease path for mean and variance since difference images and images affect the procedure. In addition, utilizing TSVD and GF with D4 does not expose ordered changes for mean, variance, and time if we regard each image pair. Furthermore, using both NLMD and NLMD with GF increases the average computing times for D2 in a noticeable way due to NLMD. Nevertheless, changes in mean and variance across all images are not in harmony, which is the case also for BF for D2.

Applying different methods generally illustrates different mean, variance, and time change effects as difference images and images are altered. Similarly, the ranking of U1 and U2 values changes depending on the image since each image has different characteristics, as mentioned in Section 3.1. Therefore, at this point, it would be more accurate to focus on the utility values obtained by considering the overall results. Because even if we use the same sensor, the dataset to be obtained may have different characteristics depending on the environment and other factors.

### 4.2. Overall Results

Table 6 presents the overall accuracy results and utility values. It shows the overall mean and variance of error metrics for each configuration considering all the images’ results. Note that employing TSVD increases the average means and decreases the average variances and computing times for D2 and D4 where averages are calculated from seven different image pair outcomes. On the other hand, GF-added configurations produce higher mean values and computing times except configuration 5, but they generate variances changing in different directions. Additionally, although NLMD shows an improvement in mean values, it gives worse results in variance and time performances for D2. On the other hand, BF increases variance values even if it displays an improvement in time and mean outcomes for D2.

Considering that we work with inputs with different characteristics, the U3 and U4 values of the overall results are considered. If there is no concern about time performance, U3 is calculated considering the high accuracy and low variance that is desired for consistency on different images and parameters. U4 is obtained when the time performance is also taken into account. Table 7 clarifies the ranking of U3 and U4 in terms of configuration number. It is apparent that using one of TSVD, NLMD, GF, or some combination thereof raises the U3 value. Additionally, a higher U3 value is obtained when the absolute value is taken, but D8 (D9 is its absolute version) is an exception. Furthermore, D6 produces a higher U3 value than D8, D4, D2, and D1 do. Difference images with more information seem to work better. As an exception, D8 (a combination of D4 and D6) give a lower U3 value than D6 but still has a close value to D6. Nevertheless, D8 generates a better U3 value than D4, D2, and D1. When we using average time calculations, of course, the rankings change.

Since there are configurations with close utility values, more than one method can be selected and applied to scoring points for the follow-up plan. For example, utility values can be normalized between 0 and 1, and those above the value obtained by subtracting a certain percentage from the highest value can be selected. As such, configurations with high accuracy and low variance are selected for a set containing data with different properties. If time performance is also important, it is also counted. After determining the U3 and U4 values that fall within a certain percentage or above the threshold value, the configurations that are common to both sets can be selected.

According to all outcomes, we can answer the questions in Section 1. We find that it is possible to decrease the sensitivity (i.e., increase consistency). On the other hand, accuracy improves while the computation time is reduced for some, but not all configurations. However, no configuration works faster than the original method (config. 1) in terms of average calculation time. Despite this, in Table 6, there are configurations with high accuracy and average time calculation values that are close to the original method’s result.

## 5. Conclusions

In this study, we compared the original PCAKM and its modified versions. All the configurations we use are deterministic, so the results are robust. In addition, none of them need the large training datasets required in supervised methods. Unsupervised methods, which work much faster than supervised methods, also stand out in terms of explainability. Today, issues such as explainability, interpretability, and transparency contribute to a trustworthy system [14], which is important for all stakeholders. Trustworthiness is an important concept to ensure that no undesirable consequences of AI systems occur during deployment.

Since PCAKM has more than one parameter combination and the analyses have different image types, it seems reasonable to look at the error metrics from an overall examination. As such, we have more consistent (i.e., less sensitive) information about the mean, variance, and time calculation performances of the error metrics. Since the difference between the error values to be obtained for all parameter combinations will be less, it will be more beneficial to use the combination of all of them. It is apparent that difference image and noise reduction makes a significant difference in the obtained results in terms of accuracy.

**Figure A2:**
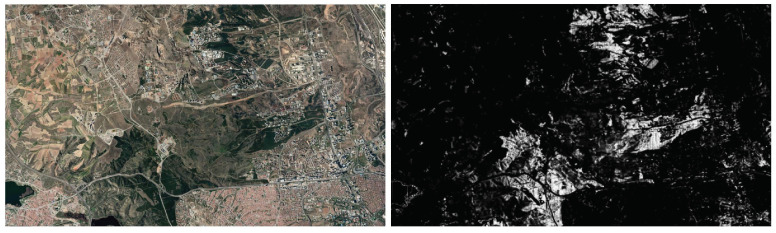
Example vegetation index result.

In the future, we plan to use the obtained results for point scoring in the follow-up activity, which may affect the road map of different agents [37,38]. A more consistent unsupervised method will help assign specific scores of interest to points on the map in a fast and efficient manner. For example, using different layer information such as the vegetation index, scoring can be done on change map information depending on the follow-up activity. In Figure A2, the figure on the left is an image taken from Google Maps and the figure on the right is the vegetation index [39] map (VIM) we calculate for this image. When a change map is produced for the image taken from Google Maps, the information to be obtained by overlapping the change map and VIM can play an important role in the scoring method according to the follow-up plan. As per the follow-up plan, VIM can belong to the first of the selected times for the change map, or it can belong to the second. In other words, it is determined by the follow-up action taken. Examples could be to investigate specific parts of the road infrastructure after an earthquake or flood.

Other maps similar to VIM can be used as labels to be illustrated on GIS. Maps that can be used for various situations (weather-related maps, information maps from the user or the planner, etc.) are merged with the change map as different layers to determine scores for the follow-up plan. Our next step will be to develop follow-up plan types and important layer maps that will contribute to the planning and scoring methods for each follow-up plan. At this point, it is worth noting the fact that SAR images are not affected by factors such as time zone and weather. Therefore, they offer an advantage in matters such as disaster response in terms of seeing the big picture. In addition, employing the proposed unsupervised method provides a robust, fast, explainable, less sensitive, and more accurate solution. These features will bridge the gap between scoring in AOIs for the follow-up plan that needs to produce a quick and estimated change map. We aim to develop an innovative scoring method for the follow-up action by merging change map results and other relevant map information as significant layers.

In addition, future work will aim to enhance the proposed change detection method with other unsupervised and supervised methods for different sensor types such as optical and thermal. In this way, the purpose of this is to obtain different layer maps by classifying the image [40]. These different layers will be used in scoring for the follow-up planning.

## Figures and Tables

**Table 1:** **Data Information.**

	Image 1 Date	Image 2 Date	Satellite	Resolution
Ottawa (Canada) [13]	May 1997	August 1997	RADARSAT	290 × 350 pixels
Yellow River Estuary 1 (China) [35]	June 2008	June 2009	RADARSAT-2	257 × 289 pixels
Yellow River Estuary 2 (China) [35]	June 2008	June 2009	RADARSAT-2	450 × 280 pixels
Yellow River Estuary 3 (China) [35]	June 2008	June 2009	RADARSAT-2	291 × 444 pixels
Yellow River Estuary 4 (China) [35]	June 2008	June 2009	RADARSAT-2	306 × 291 pixels
San Francisco (USA) [13]	August 2003	May 2004	ERS-2	256 × 256 pixels
Bern (Switzerland) [36]	April 1999	May 1999	ERS-2	301 × 301 pixels

**Table 2:** **Noise Variance Values.**

	Noise Variance Value	
	Image 1	Image 2
Ottawa	11.5067	9.0350
Yellow River Estuary 1	18.6691	37.6355
Yellow River Estuary 2	6.0765	12.5834
Yellow River Estuary 3	9.9969	26.2616
Yellow River Estuary 4	15.5373	32.9121
San Francisco	2.6013	2.8143
Bern	8.2991	7.0199

**Table 3:** **Configurations.**

No	Configuration before PCAKM	No	Configuration before PCAKM
1	$D_{1}$	12	$D_{4}$ + TSVD(var = 0.9)
2	$D_{2}$	13	GF(r = bs, ϵ = 0.0001) + $D_{4}$ + TSVD(var = 0.9)
3	$D_{2}$ + TSVD(var = 0.9)	14	$D_{5}$
4	GF(r = bs, ϵ = 0) + $D_{2}$	15	$D_{6}$
5	NLMD(h = 20, tws = 7, sws = 21) +$D_{2}$	16	GF(r = bs, ϵ = 0.0001) + $D_{6}$
6	NLMD(h = 20, tws = 7, sws = 21) + GF(r = bs, ϵ = 0) + $D_{2}$	17	$D_{7}$
7	BF(dws = 10) + $D_{2}$	18	$D_{8}$
8	$D_{3}$	19	GF(r = bs, ϵ = 0.0001) + $D_{8}$
9	$D_{4}$	20	$D_{9}$
10	GF(r = bs, ϵ = 0.0001) + $D_{4}$	21	$D_{10}$
11	$D_{4}$ + TSVD(var = 0.8)	22	$D_{11}$

**Table 4:** **Confusion Matrix.**

		Calculated Change Map	
	Pixel	*Positive* *(changed)*	*Negative* *(unchanged)*
Ground Truth Image	*Positive (changed)*	True Positive	False negative (Type II Error)
	*Negative (unchanged)*	False positive (Type I Error)	True negative

**Table 5:** **Ranking of Utility Values for Image Pairs.**

	U1	U2
Ottawa	22,21,1,12,11,3,7,13,10,16,19, 8,2,18,20,15,17,14,4,9,5,6	21,22,1,18,19,11,12,20,14,3,15, 7,2,10,8,13,17,4,16,9,5,6
Yellow River Estuary 1	7,8,3,2,22,6,5,21,19,18,17, 20,12,4,14,9,16,15,13,10,11,1	3,2,7,8,22,18,21,19,20,14,4, 11,9,10,15,12,13,17,16,6,5,1
Yellow River Estuary 2	13,11,10,19,16,12,14,9,4,20,18, 17,15,6,5,1,21,7,22,2,8,3	11,13,19,10,12,16,14,18,20,9,4, 17,15,1,6,5,21,7,22,2,8,3
Yellow River Estuary 3	16,15,17,19,20,18,10,9,4,14, 12,13,11,21,22,1,5,6,7,3,2,8	19,18,20,17,15,16,9,14,11,10,12, 4,13,21,22,1,7,8,3,2,6,5
Yellow River Estuary 4	7,5,6,3,2,8,16,15,17,19,18, 20,13,11,10,12,4,9,14,21,22,1	11,12,18,10,9,14,19,20,15,7,13, 17,8,3,21,2,16,4,22,6,5,1
San Francisco	7,5,6,3,2,8,17,15,16,18,20, 1,19,9,14,11,13,12,10,4,21,22	7,8,3,15,2,17,16,18,20,1,6, 5,19,11,14,9,12,10,13,4,21,22
Bern	7,5,6,3,8,2,16,17,15,19,18, 20,11,13,10,9,4,14,12,21,1,22	7,11,15,14,12,16,17,18,19,20,3, 8,13,9,2,10,4,21,6,5,1,22

**Table 6:** **Utility Values Based on Overall Accuracy Results and Average Computing Times for Each Configuration.**

	kc		fmeas		Avg. Time	U3	U4
No	Mean	Variance	Mean	Variance			
1	0.3599	0.0251	0.4197	0.0191	1.8071	0.7354	0.4070
2	0.5478	0.0133	0.5727	0.0106	2.1058	1.0966	0.5207
3	0.5536	**0.0122**	0.5779	0.0097	2.0302	1.1096	0.5465
4	0.5875	0.0461	0.6105	0.0429	2.1570	1.1091	0.5142
5	0.5981	0.0317	0.6128	0.0277	2.9567	1.1515	0.3895
6	0.5767	0.0364	0.6022	0.0310	2.9255	1.1115	0.3800
7	0.5839	0.0245	0.6071	0.0212	1.9838	1.1453	0.5773
8	0.5479	0.0132	0.5728	**0.0095**	2.0407	1.0980	0.5380
9	0.6155	0.0404	0.6377	0.0373	1.9563	1.1755	0.6009
10	0.6207	0.0378	0.6428	0.0348	1.9834	1.1909	0.6004
11	0.6283	0.0337	0.6491	0.0314	1.8073	1.2123	0.6708
12	0.6193	0.0369	0.6404	0.0345	1.9096	1.1884	0.6223
13	0.6294	0.0346	0.6505	0.0321	2.0829	1.2132	0.5825
14	0.6168	0.0400	0.6389	0.0369	1.8512	1.1788	0.6368
15	0.6529	0.0338	0.6737	0.0313	1.9178	1.2616	0.6578
16	**0.6630**	0.0353	**0.6837**	0.0326	2.0361	1.2788	0.6281
17	0.6541	0.0320	0.6748	0.0297	1.9922	1.2672	0.6361
18	0.6373	0.0396	0.6588	0.0365	1.8300	1.2200	0.6666
19	0.6410	0.0390	0.6624	0.0358	1.8609	1.2286	0.6602
20	0.6363	0.0398	0.6579	0.0367	1.8909	1.2176	0.6439
21	0.5240	0.0571	0.5587	0.0459	1.9765	0.9797	0.4957
22	0.4777	0.0547	0.5152	0.0435	2.0672	0.8947	0.4328

**Table 7:** **Ranking of Utility Values for Overall Results.**

	U3	U4
Overall Results	16,17,15,19,18,20,13,11,10,12, 14,9,5,7,6,3,4,8,2,21,22,1	11,18,19,15,20,14,17,16,12,9, 10,13,7,3,8,2,4,21,22,1,5,6

## Data Availability

The data presented in this study are available upon request from the corresponding author.

## Abbreviations

The following abbreviations are used in this manuscript:

AOI	Area of interest
BF	Bilateral filter
bs	Block size
c	Number of clusters
config.	Configuration
CWNN	Convolutional-wavelet neural network
DBN	Deep belief network
DCNet	Deep cascade network
dws	Denoising window size
DWT	Discrete wavelet transform
fmeas	F-measure
FNLMF	Fast non-local mean filter
FN	False negative
FP	False positive
gΓ-DBN	Gamma deep belief network
GaborFCM	Gabor fuzzy c-means
GaborPCANet	Gabor PCA network
GaborTLC	Gabor two-layer classifier
GF	Guided filter
GKSNet	Graph-based knowledge supplement network
G-MAP	Gaussian model adaptive processing
JDBN	Joint deep belief network
KC	Kappa coefficient
LR-CNN	Local restricted convolutional neural network
MLFN	Multilevel fusion network
MRFFCM	Markov random field fuzzy c-means
NFM	Non-negative matrix factorization
NLMD	Non-local means denoising
NLMF	Non-local mean filter
NLR	Nakagami log-ratio
NR-ELM	Neighborhood-based ratio and extreme learning machine
PCAKM	Principal component analysis and k-means clustering
PCC	Percentage correct classifications
PDE	Partial differential equation
PREC	Precision
SAR	Synthetic aperture radar
sws	Search windows size
TN	True negative
TP	True positive
TSVD	Truncated singular value decomposition
tws	Template windows size
VIM	Vegetation index map

## Appendix A

In this section, individual results for each image pair are given. Tables include best and worst results, mean, variance, average computing time for 1000 experiments, and utility values for each configuration. The highest best results and means, and lowest variance estimations for error metrics are shown by bold numbers.

**Table A1:** **Results of Ottawa Data.**

	Best Results			Worst Results			Mean	Var.	Avg. Time	U1	U2
No	bs	c	kc	bs	c	kc	kc	kc			
			fmeas			fmeas	fmeas	fmeas			
1	3	2	0.7684	8	2	0.6412	0.6935	**0.0011**	1.6953	1.4308	0.8440
	3	2	0.8042	8	3	0.6989	0.7392	**0.0008**			
2	3	2	0.8933	2	3	0.3077	0.6739	0.0316	1.8004	1.3319	0.7398
	3	2	0.9097	2	3	0.3832	0.7155	0.0259			
3	3	2	0.8961	2	3	0.3240	0.6912	0.0250	1.7985	1.3768	0.7655
	3	2	0.9121	2	3	0.3986	0.7310	0.0204			
4	3	2	0.8320	7	3	0.4353	0.6553	0.0171	1.8234	1.3205	0.7242
	3	2	0.8554	7	3	0.4940	0.6968	0.0145			
5	3	2	**0.8997**	2	3	0.2945	0.6526	0.0415	2.9328	1.2734	0.4342
	3	2	**0.9154**	2	3	0.3663	0.6963	0.0340			
6	3	2	**0.8997**	7	2	0.0880	0.5656	0.0731	2.8794	1.0570	0.3671
	3	2	**0.9154**	7	2	0.2279	0.6213	0.0568			
7	3	2	0.8984	2	3	0.3062	0.6914	0.0270	1.8112	1.3731	0.7581
	3	2	0.9141	2	3	0.3800	0.7309	0.0222			
8	3	2	0.8933	2	3	0.3176	0.6745	0.0311	1.8143	1.3338	0.7352
	3	2	0.9096	2	3	0.3919	0.7159	0.0255			
9	3	2	0.8321	7	3	0.4368	0.6454	0.0198	1.8124	1.2965	0.7153
	3	2	0.8555	7	3	0.4955	0.6878	0.0169			
10	3	2	0.8341	8	3	0.4422	0.6692	0.0137	1.8295	1.3534	0.7398
	3	2	0.8573	8	3	0.5024	0.7095	0.0116			
11	3	2	0.8379	8	3	0.5598	0.6896	0.0088	1.7403	1.4006	0.8048
	3	2	0.8612	8	3	0.6073	0.7274	0.0076			
12	3	2	0.8351	8	3	0.5603	0.6896	0.0080	1.7555	1.4022	0.7987
	3	2	0.8583	8	3	0.6077	0.7275	0.0069			
13	3	2	0.8355	2	3	0.4273	0.6782	0.0122	1.8816	1.3729	0.7296
	3	2	0.8587	2	3	0.4878	0.7174	0.0105			
14	3	2	0.8321	7	3	0.4367	0.6556	0.0170	1.6853	1.3212	0.7840
	3	2	0.8555	7	3	0.4953	0.6971	0.0145			
15	3	2	0.8558	2	3	0.3735	0.6624	0.0223	1.7328	1.3254	0.7649
	3	2	0.8766	2	3	0.4397	0.7039	0.0186			
16	3	2	0.8567	2	3	0.3647	0.6739	0.0192	1.8836	1.3528	0.7182
	3	2	0.8773	2	3	0.4310	0.7143	0.0162			
17	3	2	0.8558	2	3	0.3681	0.6621	0.0225	1.8266	1.3244	0.7251
	3	2	0.8766	2	3	0.4350	0.7036	0.0188			
18	3	2	0.8479	2	3	0.4013	0.6609	0.0199	1.6252	1.3265	0.8162
	3	2	0.8695	2	3	0.4642	0.7023	0.0168			
19	3	2	0.8481	2	3	0.3910	0.6714	0.0173	1.6587	1.3513	0.8147
	3	2	0.8698	2	3	0.4545	0.7118	0.0146			
20	3	2	0.8479	2	3	0.4006	0.6608	0.0199	1.6872	1.3263	0.7861
	3	2	0.8696	2	3	0.4636	0.7022	0.0168			
21	3	2	0.8850	8	3	0.7041	0.7970	0.0025	1.7493	1.6208	0.9265
	3	2	0.9037	8	3	0.7458	0.8282	0.0019			
22	3	3	0.8899	8	3	0.7220	**0.7985**	0.0021	1.8765	1.6253	0.8661
	3	3	0.9067	8	3	0.7627	**0.8304**	0.0015			

**Table A2:** **Results of Yellow River Estuary 1.**

	Best Results			Worst Results			Mean	Var.	Avg. Time	U1	U2
No	bs	c	kc	bs	c	kc	kc	kc			
			fmeas			fmeas	fmeas	fmeas			
1	3	3	0.4159	7	2	−0.3118	0.0061	0.0856	1.5046	0.0808	0.0537
	3	3	0.5293	8	3	0	0.2174	0.0571			
2	5	2	0.8183	8	3	0.3074	0.7049	0.0250	1.5639	1.4120	0.9029
	5	2	0.8516	8	3	0.3989	0.7519	0.0198			
3	5	2	0.8183	8	3	0.3126	0.7080	0.0248	1.5519	1.4179	0.9137
	5	2	0.8516	8	3	0.4031	0.7544	0.0197			
4	5	2	0.7638	2	3	0.4219	0.5940	0.0147	1.5725	1.2127	0.7712
	7	2	0.8035	2	3	0.4759	0.6466	0.0132			
5	3	2	0.8261	6	3	0.2404	0.6821	0.0443	2.3293	1.3326	0.5721
	3	2	0.8572	6	3	0.3318	0.7304	0.0356			
6	3	2	0.8259	6	3	0.2404	0.6824	0.0443	2.3099	1.3333	0.5772
	3	2	0.8571	6	3	0.3318	0.7308	0.0356			
7	3	2	**0.8390**	8	3	0.2845	**0.7280**	0.0327	1.5973	1.4408	0.9020
	3	2	**0.8679**	8	3	0.3776	**0.7714**	0.0259			
8	5	2	0.8183	8	3	0.3070	0.7051	0.0250	1.6022	1.4202	0.8864
	5	2	0.8516	8	3	0.3986	0.7521	0.0120			
9	5	2	0.7638	2	3	0.4222	0.5927	0.0150	1.5987	1.2096	0.7566
	7	2	0.8035	2	3	0.4762	0.6453	0.0134			
10	5	2	0.7656	8	3	0.4092	0.5892	0.0166	1.6074	1.2004	0.7468
	7	2	0.8041	2	3	0.4832	0.6424	0.0146			
11	5	2	0.7629	6	3	0.4088	0.5831	0.0183	1.5654	1.1849	0.7569
	7	2	0.8016	6	3	0.4776	0.6360	0.0159			
12	5	2	0.7633	2	3	0.4369	0.5957	0.0147	1.6521	1.2154	0.7357
	7	2	0.8037	2	3	0.4904	0.6476	0.0132			
13	5	2	0.7639	8	3	0.4013	0.5898	0.0170	1.7313	1.2004	0.6934
	7	2	0.8049	8	3	0.4776	0.6425	0.0149			
14	5	2	0.7638	2	3	0.4219	0.5931	0.0149	1.5611	1.2106	0.7755
	7	2	0.8037	2	3	0.4759	0.6457	0.0133			
15	5	2	0.7755	6	3	0.3777	0.5926	0.0204	1.6121	1.2028	0.7461
	5	2	0.8132	6	3	0.4527	0.6478	0.0172			
16	5	2	0.7775	6	3	0.3804	0.5944	0.0204	1.8577	1.2061	0.6492
	5	2	0.8149	5	3	0.4542	0.6493	0.0172			
17	5	2	0.7755	6	3	0.3777	0.6014	0.0181	1.8254	1.2234	0.6702
	5	2	0.8132	6	3	0.4527	0.6555	0.0154			
18	5	2	0.7698	5	3	0.4320	0.6067	0.0140	1.5299	1.2396	0.8102
	7	2	0.8085	5	3	0.4973	0.6592	0.0123			
19	5	2	0.7721	5	3	0.4357	0.6089	0.0139	1.5428	1.2440	0.8063
	5	2	0.8101	5	3	0.5001	0.6612	0.0122			
20	5	2	0.7698	6	3	0.3876	0.5979	0.0167	1.5619	1.2181	0.7799
	7	2	0.8084	6	3	0.4610	0.6514	0.0145			
21	5	2	0.7599	8	3	0.3010	0.6401	0.0213	1.6163	1.3040	0.8068
	7	2	0.8063	8	3	0.3995	0.7013	0.0161			
22	7	2	0.7470	2	2	0.4690	0.6762	**0.0067**	1.7252	1.4023	0.8128
	7	2	0.7980	2	2	0.5907	0.7365	**0.0037**			

**Table A3:** **Results of Yellow River Estuary 2.**

	Best Results			Worst Results			Mean	Var.	Avg. Time	U1	U2
No	bs	c	kc	bs	c	kc	kc	kc			
			fmeas			fmeas	fmeas	fmeas			
1	4	3	0.1755	8	2	0.0753	0.1136	0.0014	2.0835	0.2417	0.1160
	4	3	0.1906	8	2	0.0940	0.1308	0.0013			
2	2	3	−0.0170	7	2	−0.0211	−0.0200	$1.22\times 10^{-6}$	3.4239	−0.0188	−0.0055
	2	3	0.00358	7	2	0.0003	0.0012	$8.32\times 10^{-7}$			
3	2	2	−0.0186	7	2	−0.0211	−0.0201	$7.09\times 10^{-7}$	3.0496	−0.0191	−0.0063
	2	2	0.00257	7	2	0.0003	0.0010	$4.79\times 10^{-7}$			
4	8	3	0.8027	5	3	0.0308	0.4356	0.1224	3.4543	0.6418	0.1858
	8	3	0.8047	5	3	0.0500	0.4456	0.1170			
5	7	3	0.7073	8	2	−0.0206	0.2478	0.1006	3.9585	0.3123	0.0789
	7	3	0.7111	8	2	0	0.2614	0.0963			
6	7	3	0.7100	8	2	−0.0206	0.2488	0.1013	3.9399	0.3130	0.0794
	7	3	0.7137	8	2	0	0.2624	0.0969			
7	7	3	0.6588	3	2	−0.0212	0.1221	0.0746	3.2626	0.1158	0.0355
	7	3	0.6635	2	3	0	0.1394	0.0711			
8	2	3	−0.0169	7	2	−0.0211	−0.0200	$1.22\times 10^{-6}$	3.2725	−0.0189	−0.0058
	2	3	0.0036	7	2	0.0003	0.0011	$8.30\times 10^{-7}$			
9	8	3	0.8031	5	3	0.0362	0.4366	0.1216	3.1164	0.6454	0.2071
	8	3	0.8050	5	3	0.0553	0.4466	0.1162			
10	6	2	0.8063	5	2	0.0770	0.4897	0.1047	3.1475	0.7834	0.2489
	6	2	0.8083	5	2	0.0958	0.4985	0.1001			
11	6	2	0.8145	5	2	0.0786	0.5271	0.0942	2.6834	0.8778	0.3271
	6	2	0.8164	5	2	0.0973	0.5349	0.0900			
12	6	2	0.8058	5	2	0.0742	0.4472	0.1144	2.8518	0.6803	0.2386
	6	2	0.8078	5	2	0.0931	0.4569	0.1094			
13	6	2	**0.8146**	5	2	0.0843	**0.5300**	0.0922	3.0858	0.8875	0.2876
	6	2	**0.8165**	5	2	0.1028	**0.5378**	0.0881			
14	8	3	0.8027	5	3	0.0328	0.4367	0.1216	2.7668	0.6456	0.2333
	8	3	0.8047	5	3	0.0520	0.4467	0.1162			
15	6	2	0.8097	3	3	0.0340	0.3826	0.1418	2.9570	0.4994	0.1689
	6	2	0.8117	3	3	0.0529	0.3941	0.1355			
16	6	2	0.8109	5	2	0.0604	0.4822	0.1284	3.0952	0.7225	0.2334
	6	2	0.8129	5	2	0.0797	0.4913	0.1226			
17	6	2	0.8097	3	3	0.0326	0.3826	0.1318	3.378	0.5190	0.1708
	6	2	0.8117	3	3	0.0516	0.3941	0.1259			
18	6	2	0.8099	3	3	0.0368	0.4311	0.1293	2.7983	0.6196	0.2214
	6	2	0.8119	3	3	0.0555	0.4414	0.1236			
19	6	2	0.8113	5	2	0.0652	0.4832	0.1147	2.8843	0.7513	0.2605
	6	2	0.8133	5	2	0.0843	0.4923	0.1095			
20	6	2	0.8099	3	3	0.0368	0.4328	0.1284	2.9458	0.6249	0.2121
	6	2	0.8119	3	3	0.0555	0.4431	0.1226			
21	8	3	0.7953	2	2	0.0265	0.0929	0.0380	3.1092	0.1301	0.0418
	8	3	0.7976	2	2	0.0468	0.1115	0.0363			
22	8	3	0.0563	2	3	−0.0116	0.0337	0.0003	3.2598	0.0868	0.0266
	8	3	0.0757	2	3	0.0091	0.0537	0.0003			

**Table A4:** **Results of Yellow River Estuary 3.**

	Best Results			Worst Results			Mean	Var.	Avg. Time	U1	U2
No	bs	c	kc	bs	c	kc	kc	kc			
			fmeas			fmeas	fmeas	fmeas			
1	7	3	0.3469	3	2	0.0926	0.2145	0.0085	2.6281	0.4567	0.1738
	7	3	0.3790	3	2	0.1473	0.2577	0.0070			
2	5	3	0.3124	6	2	−0.0100	0.1443	0.0236	2.7941	0.2837	0.1015
	5	3	0.3224	6	2	0.0501	0.1796	0.0166			
3	5	3	0.3114	3	2	−0.0105	0.1446	0.0237	2.7767	0.2840	0.1023
	5	3	0.3213	3	2	0.0497	0.1798	0.0167			
4	5	2	0.7622	8	3	0.5946	0.6834	0.0023	2.8094	1.3713	0.4881
	5	2	0.7697	8	3	0.6048	0.6925	0.0023			
5	5	3	0.3163	8	2	0.0018	0.1548	0.0230	4.0698	0.3041	0.0747
	5	3	0.3256	8	2	0.0599	0.1886	0.0163			
6	3	3	0.3190	6	2	0.0014	0.1547	0.0231	4.0529	0.3038	0.0750
	3	3	0.3292	6	2	0.0596	0.1886	0.0164			
7	3	3	0.3245	3	2	−0.0066	0.1524	0.0250	2.5647	0.2966	0.1156
	3	3	0.3345	3	2	0.0529	0.1870	0.0178			
8	5	3	0.3124	6	2	−0.0104	0.1442	0.0236	2.6530	0.2835	0.1069
	5	3	0.3224	5	2	0.0498	0.1795	0.0166			
9	5	2	0.7618	8	3	0.5946	0.6840	0.0023	2.6035	1.3724	0.5271
	5	2	0.7693	8	3	0.6048	0.6930	0.0023			
10	5	2	0.7658	8	3	0.5831	0.6845	0.0027	2.6736	1.3726	0.5134
	5	2	0.7731	8	3	0.5933	0.6934	0.0026			
11	5	2	0.7635	8	3	0.5294	0.6596	0.0057	2.5456	1.3169	0.5173
	5	2	0.7708	8	3	0.5398	0.6686	0.0056			
12	5	2	0.7638	8	3	0.5879	0.6800	0.0028	2.7567	1.3634	0.4946
	5	2	0.7712	8	3	0.5981	0.6890	0.0028			
13	5	2	0.7681	8	3	0.5601	0.6770	0.0037	3.0288	1.3556	0.4476
	5	2	0.7754	8	3	0.5704	0.6860	0.0037			
14	5	2	0.7618	8	3	0.5946	0.6835	0.0024	2.6143	1.3713	0.5245
	5	2	0.7693	8	3	0.6048	0.6925	0.0023			
15	5	2	0.7686	8	3	0.6731	0.7118	**0.0008**	2.6357	1.4310	0.5429
	5	2	0.7763	8	3	0.6829	0.7208	**0.0008**			
16	5	2	**0.7729**	8	3	0.6743	**0.7151**	**0.0008**	2.6496	1.4375	0.5425
	5	2	**0.7804**	8	3	0.6840	**0.7240**	**0.0008**			
17	5	2	0.7686	8	3	0.6728	0.7117	**0.0008**	2.6353	1.4308	0.5429
	5	2	0.7763	8	3	0.6826	0.7207	**0.0008**			
18	5	2	0.7658	8	3	0.6538	0.7040	0.0010	2.4470	1.4150	0.5783
	5	2	0.7733	8	3	0.6638	0.7130	0.0010			
19	5	2	0.7689	8	3	0.6459	0.7062	0.0011	2.4506	1.4191	0.5791
	5	2	0.7763	8	3	0.6557	0.7151	0.0011			
20	5	2	0.7658	8	3	0.6538	0.7041	0.0010	2.5109	1.4152	0.5636
	5	2	0.7733	8	3	0.6638	0.7131	0.0010			
21	7	3	0.7148	2	2	0.1612	0.4862	0.0342	2.6083	0.9339	0.3580
	7	3	0.7255	2	2	0.2103	0.5115	0.0296			
22	7	3	0.6692	2	2	0.1264	0.4094	0.0386	2.6198	0.7777	0.2969
	7	3	0.6825	2	2	0.1786	0.4402	0.0333			

**Table A5:** **Results of Yellow River Estuary 4.**

	Best Results			Worst Results			Mean	Var.	Avg. Time	U1	U2
No	bs	c	kc	bs	c	kc	kc	kc			
			fmeas			fmeas	fmeas	fmeas			
1	6	3	0.6450	2	2	0.2341	0.4396	0.0228	1.7555	0.8878	0.5057
	6	3	0.6691	2	2	0.3094	0.4884	0.0174			
2	6	3	0.8563	8	2	0.6501	0.7611	0.0049	2.0176	1.5295	0.7581
	6	3	0.8646	8	2	0.6771	0.7774	0.0041			
3	6	3	0.8558	8	2	0.6536	0.7660	**0.0042**	1.9845	1.5402	0.7761
	6	3	0.8642	8	2	0.6803	0.7819	**0.0035**			
4	6	2	0.8431	7	3	0.5453	0.7442	0.0084	2.1660	1.4851	0.6856
	6	2	0.8523	7	3	0.5649	0.7571	0.0078			
5	6	3	0.8677	8	2	0.6288	**0.7745**	0.0063	2.8495	1.5530	0.5450
	6	3	0.8755	8	2	0.6580	**0.7900**	0.0052			
6	6	3	0.8676	8	2	0.6288	0.7744	0.0063	2.8352	1.5528	0.5477
	6	3	0.8754	8	2	0.6580	0.7899	0.0052			
7	3	3	**0.8681**	8	2	0.6494	0.7741	0.0055	1.9147	1.5537	0.8115
	3	3	**0.8758**	8	2	0.6766	0.7897	0.0046			
8	6	3	0.8564	8	2	0.6501	0.7610	0.0049	1.9416	1.5293	0.7876
	6	3	0.8647	8	2	0.6771	0.7773	0.0041			
9	6	2	0.8433	7	3	0.5453	0.7442	0.0084	1.7349	1.4851	0.8560
	6	2	0.8525	7	3	0.5649	0.7571	0.0078			
10	6	2	0.8445	7	3	0.5461	0.7453	0.0084	1.7355	1.4872	0.8569
	6	2	0.8536	7	3	0.5656	0.7581	0.0078			
11	6	2	0.8415	7	3	0.5500	0.7455	0.0083	1.6289	1.4874	0.9131
	6	2	0.8508	7	3	0.5691	0.7580	0.0078			
12	6	2	0.8431	8	3	0.5473	0.7444	0.0084	1.7001	1.4853	0.8737
	6	2	0.8523	7	3	0.5674	0.7572	0.0079			
13	6	2	0.8441	7	3	0.5478	0.7493	0.0084	1.8793	1.4916	0.7937
	6	2	0.8532	7	3	0.5673	0.7586	0.0079			
14	6	2	0.8433	7	3	0.5453	0.7438	0.0084	1.7351	1.4842	0.8554
	6	2	0.8525	7	3	0.5649	0.7567	0.0079			
15	6	2	0.8456	7	3	0.5290	0.7528	0.0094	1.8070	1.5005	0.8304
	6	2	0.8549	7	3	0.5513	0.7658	0.0087			
16	6	2	0.8461	8	3	0.5309	0.7539	0.0094	1.9894	1.5026	0.7553
	6	2	0.8553	7	3	0.5522	0.7668	0.0087			
17	6	2	0.8456	7	3	0.5278	0.7527	0.0095	1.8993	1.5000	0.7898
	6	2	0.8549	7	3	0.5491	0.7656	0.0088			
18	6	2	0.8440	8	3	0.5373	0.7504	0.0089	1.7354	1.4965	0.8623
	6	2	0.8533	7	3	0.5582	0.7633	0.0083			
19	6	2	0.8448	7	3	0.5371	0.7510	0.0090	1.7527	1.4976	0.8545
	6	2	0.8540	7	3	0.5575	0.7639	0.0083			
20	6	2	0.8438	8	3	0.5373	0.7503	0.0089	1.7641	1.4963	0.8482
	6	2	0.8531	7	3	0.5582	0.7632	0.0083			
21	6	3	0.8431	2	2	0.3453	0.6858	0.0198	1.7682	1.3595	0.7689
	6	3	0.8521	2	2	0.4061	0.7094	0.0159			
22	6	3	0.8335	2	2	0.2476	0.5938	0.0376	1.7717	1.1538	0.6512
	6	3	0.8434	2	2	0.3216	0.6276	0.0300			

**Table A6:** **Results of San Francisco.**

	Best Results			Worst Results			Mean	Var.	Avg. Time	U1	U2
No	bs	c	kc	bs	c	kc	kc	kc			
			fmeas			fmeas	fmeas	fmeas			
1	8	3	0.7321	2	2	0.4188	0.5555	0.0145	1.1227	1.1278	1.0045
	8	3	0.7530	2	2	0.4797	0.5977	0.0109			
2	5	3	0.9054	2	2	0.8226	0.8691	0.0005	1.2129	1.7472	1.4405
	5	3	0.9122	2	2	0.8368	0.8790	0.0004			
3	3	3	0.9067	8	2	0.8566	0.8830	**0.0002**	1.1985	1.7502	1.4603
	3	3	0.9133	8	2	0.8676	0.8676	**0.0002**			
4	5	2	0.8738	3	2	−0.0680	0.3697	0.1452	1.3166	0.4931	0.3745
	5	2	0.8825	3	2	0.0000	0.4017	0.1331			
5	2	3	**0.9157**	8	2	0.8580	0.8860	0.0003	1.8290	1.7800	0.9732
	2	3	**0.9219**	8	2	0.8688	**0.8946**	0.0003			
6	3	3	0.9151	8	2	0.8592	0.8858	0.0003	1.8041	1.7797	0.9865
	3	3	0.9213	8	2	0.8700	0.8944	**0.0002**			
7	3	3	0.9122	8	2	0.8594	**0.8862**	**0.0002**	1.1413	1.7804	1.5600
	3	3	0.9185	8	2	0.8701	**0.8946**	**0.0002**			
8	5	3	0.9054	2	2	0.8226	0.8690	0.0005	1.1414	1.7471	1.5307
	5	3	0.9122	2	2	0.8368	0.8790	0.0004			
9	5	2	0.8738	7	3	0.1974	0.5755	0.1032	1.1382	0.9809	0.8618
	5	2	0.8825	7	3	0.2421	0.6008	0.0922			
10	5	2	0.8762	7	3	0.1983	0.5351	0.1064	1.1416	0.8962	0.7850
	5	2	0.8848	7	3	0.2428	0.5626	0.0951			
11	3	2	0.8654	8	3	0.2306	0.5547	0.0934	1.0069	0.9522	0.9457
	3	2	0.8744	7	3	0.2662	0.5768	0.0859			
12	5	2	0.8736	7	3	0.2128	0.5493	0.0974	1.1211	0.9360	0.8349
	5	2	0.8822	7	3	0.2509	0.5729	0.0888			
13	5	2	0.8751	7	3	0.2137	0.5516	0.0967	1.2936	0.9417	0.7280
	5	2	0.8836	7	3	0.2522	0.5750	0.0882			
14	5	2	0.8738	3	3	0.1973	0.5751	0.1030	1.0704	0.9804	0.9159
	5	2	0.8825	7	3	0.2421	0.6004	0.0921			
15	7	3	0.8763	8	3	0.1782	0.8120	0.0311	1.0874	1.5780	1.4512
	7	3	0.8849	8	3	0.2274	0.8247	0.0276			
16	5	2	0.8779	7	3	0.1630	0.7626	0.0585	1.1573	1.4302	1.2358
	5	2	0.8865	7	3	0.2129	0.7781	0.0520			
17	7	3	0.8763	8	3	0.1782	0.8121	0.0311	1.1042	1.5782	1.4293
	7	3	0.8849	8	3	0.2274	0.8248	0.0276			
18	5	2	0.8759	5	3	0.1700	0.6636	0.0930	1.0593	1.1724	1.1068
	5	2	0.8846	5	3	0.2202	0.6846	0.0828			
19	5	2	0.8773	4	3	0.1713	0.6185	0.1060	1.0963	1.0603	0.9672
	5	2	0.8859	7	3	0.2219	0.6421	0.0943			
20	5	2	0.8759	5	3	0.1700	0.6636	0.0930	1.1099	1.1724	1.0563
	5	2	0.8846	5	3	0.2202	0.6846	0.0828			
21	7	3	0.8855	8	2	−0.1361	0.3589	0.2442	1.1173	0.3651	0.3268
	7	3	0.8937	2	2	0.0034	0.4348	0.1844			
22	7	3	0.8840	8	2	−0.1355	0.3561	0.2409	1.1223	0.3651	0.3253
	7	3	0.8923	2	2	0.0032	0.4320	0.1821			

**Table A7:** **Results of Bern.**

	Best Results			Worst Results			Mean	Var.	Avg. Time	U1	U2
No	bs	c	kc	bs	c	kc	kc	kc			
			fmeas			fmeas	fmeas	fmeas			
1	5	3	0.7359	2	2	0.1204	0.4966	0.0415	1.8599	0.9222	0.4958
	5	3	0.7398	2	2	0.1412	0.5064	0.0393			
2	3	2	0.8292	6	3	0.5664	0.7014	0.0076	1.9276	1.3906	0.7214
	3	2	0.8312	6	3	0.5697	0.7044	0.0076			
3	3	2	0.8287	4	3	0.5695	0.7026	0.0076	1.8519	1.3930	0.7522
	3	2	0.8306	4	3	0.5728	0.7056	0.0076			
4	5	2	0.7763	2	3	0.4559	0.6300	0.0123	1.9566	1.2385	0.6330
	5	2	0.7788	2	3	0.4591	0.6331	0.0123			
5	3	2	0.8398	8	3	0.5763	0.7256	**0.0060**	2.7281	1.4421	0.5286
	3	2	0.8417	8	3	0.5797	0.7285	**0.0060**			
6	3	2	0.8398	8	3	0.5771	0.7250	0.0061	2.6568	1.4409	0.5423
	3	2	0.8417	8	3	0.5805	0.7280	**0.0060**			
7	3	2	**0.8509**	8	3	0.5936	**0.7333**	0.0065	1.5951	1.4566	0.9132
	3	2	**0.8527**	8	3	0.5970	**0.7362**	0.0064			
8	3	2	0.8292	6	3	0.5656	0.7018	0.0076	1.8600	1.3914	0.7481
	3	2	0.8312	6	3	0.5688	0.7048	0.0076			
9	5	2	0.7763	2	3	0.4559	0.6301	0.0124	1.6901	1.2386	0.7329
	5	2	0.7788	2	3	0.4591	0.6332	0.0123			
10	5	2	0.7771	2	3	0.4650	0.6317	0.0120	1.7489	1.2425	0.7104
	5	2	0.7796	2	3	0.4683	0.6348	0.0120			
11	7	2	0.7669	2	3	0.5362	0.6386	0.0072	1.4803	1.2659	0.8552
	7	2	0.7701	2	3	0.5394	0.6417	0.0072			
12	3	2	0.7732	2	3	0.4487	0.6286	0.0124	1.5298	1.2357	0.8078
	3	2	0.7755	2	3	0.4519	0.6318	0.0123			
13	5	2	0.7765	2	3	0.4657	0.6330	0.0117	1.6798	1.2458	0.7416
	5	2	0.7790	2	3	0.4690	0.6361	0.0116			
14	5	2	0.7763	2	3	0.4559	0.6300	0.0124	1.5257	1.2384	0.8117
	5	2	0.7788	2	3	0.4591	0.6331	0.0123			
15	3	2	0.8140	2	3	0.5152	0.6557	0.0105	1.5926	1.2935	0.8122
	3	2	0.8161	2	3	0.5185	0.6588	0.0105			
16	3	2	0.8186	2	3	0.5274	0.6585	0.0104	1.6196	1.2993	0.8022
	3	2	0.8206	2	3	0.5306	0.6616	0.0104			
17	3	2	0.8140	2	3	0.5152	0.6559	0.0105	1.6165	1.2940	0.8005
	3	2	0.8161	2	3	0.5185	0.6590	0.0104			
18	5	2	0.7905	2	3	0.4890	0.6444	0.0110	1.6152	1.2699	0.7862
	5	2	0.7929	2	3	0.4922	0.6475	0.0110			
19	3	2	0.7940	2	3	0.5086	0.6476	0.0107	1.6412	1.2770	0.7781
	3	2	0.7962	2	3	0.5118	0.6507	0.0106			
20	5	2	0.7905	2	3	0.4890	0.6444	0.0110	1.6568	1.2699	0.7665
	5	2	0.7929	2	3	0.4922	0.6475	0.0110			
21	3	3	0.8034	2	2	0.1268	0.6069	0.0394	1.8668	1.1443	0.6130
	3	3	0.8062	2	2	0.1473	0.6141	0.0373			
22	5	3	0.7615	2	2	0.0864	0.4757	0.0566	2.0952	0.8518	0.4065
	5	3	0.7650	2	2	0.1084	0.4863	0.0536			

## Appendix B

Image pairs with ground truth and best result images are illustrated in Figure A1. Since we made the images equal in size, some of them seem to be scaled according to their original versions.

Google Maps image and its vegetation index map is illustrated in Figure A2.
